# Brain–computer interface digital prescription for neurological disorders

**DOI:** 10.1111/cns.14615

**Published:** 2024-02-15

**Authors:** Xiaoke Chai, Tianqing Cao, Qiheng He, Nan Wang, Xuemin Zhang, Xinying Shan, Zeping Lv, Wenjun Tu, Yi Yang, Jizong Zhao

**Affiliations:** ^1^ Brain Computer Interface Transitional Research Center, Beijing Tiantan Hospital Capital Medical University Beijing China; ^2^ China National Center for Neurological Disorders Beijing China; ^3^ Translation Laboratory of Clinical Medicine Chinese Institute for Brain Research & Beijing Tiantan Hospital Beijing China; ^4^ Department of Neurosurgery, Beijing Tiantan Hospital Capital Medical University Beijing China; ^5^ China National Clinical Research Center for Neurological Diseases Beijing China; ^6^ National Research Center for Rehabilitation Technical Aids Beijing China; ^7^ Beijing Institute of Brain Disorders Beijing China; ^8^ Chinese Institute for Brain Research Beijing China

**Keywords:** brain–computer Interface, disorders, neural modulation, rehabilitation

## Abstract

Neurological and psychiatric diseases can lead to motor, language, emotional disorder, and cognitive, hearing or visual impairment By decoding the intention of the brain in real time, the Brain–computer interface (BCI) can first assist in the diagnosis of diseases, and can also compensate for its damaged function by directly interacting with the environment; In addition, provide output signals in various forms, such as actual motion, tactile or visual feedback, to assist in rehabilitation training; Further intervention in brain disorders is achieved by close‐looped neural modulation. In this article, we envision the future BCI digital prescription system for patients with different functional disorders and discuss the key contents in the prescription the brain signals, coding and decoding protocols and interaction paradigms, and assistive technology. Then, we discuss the details that need to be specially included in the digital prescription for different intervention technologies. The third part summarizes previous examples of intervention, focusing on how to select appropriate interaction paradigms for patients with different functional impairments. For the last part, we discussed the indicators and influencing factors in evaluating the therapeutic effect of BCI as intervention.

## Graphical abstract

BCI prescription for neurological disorders.

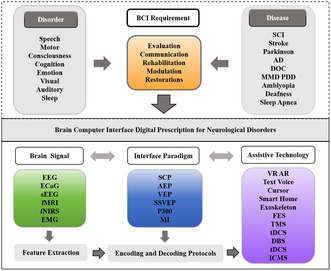

## 1 MAIN CONTENT OF BCI DIGITAL PRESCRIPTION

Brain–computer interface (BCI) technology is a multidisciplinary subject involving cognitive neuroscience, Biomedical engineering, material science, computer information technology and Applied mathematics. In recent years, the rapid development of BCI has benefits from two technological advances: one is to improve the quality of input signal source through high‐density minimally invasive electrode implantation, and the other is the application of unsupervised deep learning algorithm in brain intention recognition. This makes BCI more and more effective in the medical field, including diagnosis, rehabilitation, and treatment of brain diseases. Not only can communication with the outside world be achieved through real‐time encoding and decoding of brain signals,^1^ but more importantly, various feedback methods can achieve enhancement or restoration of sensory perception, cognitive, and motor functions.^2^

**FIGURE 1:**
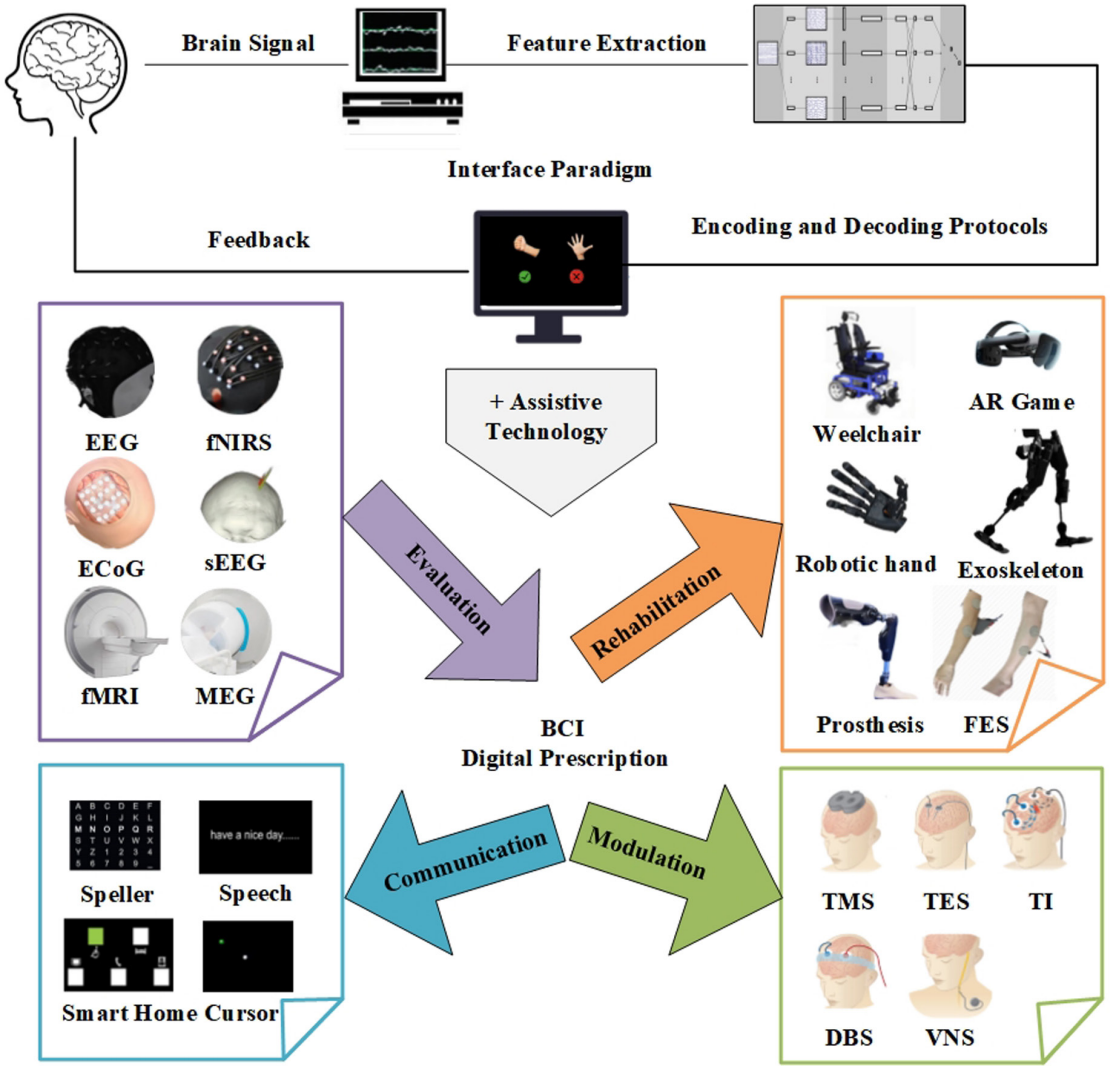
Main content of the BCI prescription including brain signal, feature extraction, control protocol, and interface paradigm for each application such as evaluation, communication, rehabilitation and modulation^14^ which combined with related Assistive technology.

Since there exist various requirements of BCI in clinical for patients, however, the standardized methods and processes for clinicians have not yet been established. In this review, the construction of a digital prescription system for BCI will be discussed. As shown in Figure 1, the provisions of BCI should include what kind of brain signal to input, feature extraction method, coding and decoding protocol, and feedback mode of the interaction paradigm. Besides those main content, BCI for the various requirements, it is also necessary to describe the combined assistive technology, as well as specific performance indicators, training plan, and other specific related details.

### 1.1 Neural signal input

BCIs can be implemented using various brain information, such as magnetoencephalography (MEG), functional magnetic resonance imaging (fMRI), near infrared spectroscopy (NIRS), and electroencephalography (EEG).^3^, ^4^, ^5^ In particular, BCIs based on non‐implantable EEG have been widely utilized due to their high safety, low cost, and ease of acquisition.^6^ For the clinics, intracranial electroencephalogram (iEEG), including EcoG and Stereo‐electroencephalogram (sEEG), is also very important in the diagnosis and evaluation of neurosurgery diseases. More and more BCI is based on implantable signals to obtain stable and high spatiotemporal resolution signals, thereby achieving high‐precision decoding of intentions.^7^

How to choose the input brain signal, whether it is invasive or non‐invasive, should depend on the application scenario and the treatment plan for the patient, as for motor function rehabilitation training, non‐invasive and portable EEG may meet most of the needs, while more precise and stable neural regulation, high‐precision cortical or deep brain signals may also be required.

### 1.2 Encoding and decoding protocol

More and more deep learning methods have been applied to classifying brain signals to improve the overall quality of pattern recognition accuracy. Especially in the BCI based on EEG signals, the application of deep learning algorithms in the encoding and decoding of BCI not only improves the recognition accuracy and efficiency in classification but also realizes two‐way information exchange based on EEG signal prediction in closed‐loop systems.^8^ However, complex deep learning algorithms not only consume training time but also have certain limitations in real‐time performance.

### 1.3 Interface paradigm

Various EEG signals have been studied to convert human purposes into control commands, among which the evoked potential P300,^9^ steady state visual evoked potential^10^, ^11^ and the motor imagery (MI)^7^ are the most common BCI paradigm. The choice of BCI paradigm depends more on the patient's functional impairment, such as for patients with disorders of consciousness, they are inability to use complex paradigms related to advanced cognition and may prefer passive perceptual paradigms.^12^ For patients who need assistance in restoring motor function as much as possible, an active BCI paradigm with feedback can better stimulate their corresponding functional brain areas. The increasing complexity of multimodal interaction paradigms may lead to a certain degree of psychological and physical fatigue, leading to a decrease in system performance.^13^

The main content of the BCI prescription should include signal input, feature extraction, encoding and decoding protocol, and interface paradigm. As in Figure 1, for each application combined with other assistive technology, more details need to be clearly described for evaluation, communication, rehabilitation and modulation^14^ of BCI digital prescriptions.

## 2 BCI PRESCRIPTION CATEGORY FOR DIFFERENT REQUIREMENTS

According to different needs and in combination with related Assistive technology, different types of BCI digital prescriptions need to include specific details. Next, it is mainly divided into the following four types, including BCI for brain function evaluation, daily communication, rehabilitation training and Neuromodulation. Evaluation BCI is designed for multi‐mode brain function diagnosis and assessment, communication BCI is for daily communication and control, Rehabilitation BCI is used for functional rehabilitation training and adaptive feedback remodeling, and neural modulation BCI is designed for network positioning and intervention. As shown in Figure 1, the application of BCI in clinical practice has gradually evolved from brain control of external devices to brain regulation through interfaces. Design interfaces with other Assistive technology according to use needs, such as combining virtual reality augmented reality to design rehabilitation training scenes and tasks, and combining various invasive and non‐invasive nerve modulation technologies, such as transcranial magnetic stimulation and cortical electrical stimulation to regulate neural function.^15^

The future clinical BCI should be aimed at patients with different diseases and dysfunction, and be able to issue corresponding standard digital prescribing. The content of the prescription should first describe their functional compensation or enhancement to be achieved by BCI‐assisted rehabilitation training or closed‐loop stimulation modulation. The digital prescribing provides specific instructions on which signals are suitable for patients to use as BCI inputs, which interaction modes are designed, what are the corresponding encoding and decoding modes, how long the training will take, as well as the training cycle and expected implementation.

### 2.1 Assistant technology in evaluation BCI prescription

In recent years, the rise of deep learning has greatly improved the accuracy of BCI in cognitive impairment assessment and early recognition. And there has also been breakthrough progress in the pre seizure warning of epilepsy.^16^ As for the BCI for diagnostic evaluation, the input signals are mostly non‐invasive and has multiple mode, the key design is to decide the psychological paradigm corresponding to its corresponding cognitive impairment, then obtain the accuracy of classification or prediction based on specific diagnostic indicators.

### 2.2 Assistant technology in communication BCI prescription

BCI were first used in the field of neural engineering for communication control between the brain and the external environment, such as helping amputees, stroke or severely paralyzed patients with spinal cord injury operate wheelchairs, walking aids, exoskeletons, and robotic arms to assist their daily lives. In recent years, more research has focused on active language communication rather than passive speller.^17^, ^18^, ^19^ The BCI prescription for communication needs more explanation of the design in the number and type of control commands, as well as the degree of acceptance of delay, reliable interaction schemes and corresponding encoding and decoding modes, all those in order to balance the performance and workload.

### 2.3 Assistant technology in rehabilitation BCI prescription

BCI has been applied to the rehabilitation of upper and lower limbs, and research has shown that physical therapy combined with BCI technology has significantly better therapeutic effects in limb function recovery compared with the traditional treatment plans. The principle of this technology is to use the principle of neuroplasticity, through different feedback methods to assist training, and code the command of the training exoskeleton by identifying the movement intention.^20^

With the continuous development of BCI, more intelligent rehabilitation treatment methods can be widely applied to patients with functional disorders. The BCI system can record cortical activities and extract information related to expected functions, transmit action commands to external devices, and through BCI, combine function electronic stimulation (FES), virtual reality (VR), and dynamic Exoskeleton, it can truly realize synchronous coupling of cortical and muscular activities.^21^ So as to promote the reconstruction of patients' personalized Neural circuits and personalized training to promote the rehabilitation of limbs and limb motor function. In this kind of the BCI prescription, it is more necessary to explain the training scheme and the design of the feedback mode that is helpful for the recovery of limb function.

### 2.4 Assistant Technology in modulation BCI prescription

At present, BCI has been applied to the non‐invasive and invasive biofeedback system, which relies on the rapid processing of brain signals. The closed‐loop real‐time BCI technology, combined with various types of electrical and magnetic stimulation technologies, has been applied to the modulation of depression, epilepsy, and diseases such as Parkinson's, which can reduce seizures and improve emotions and attention. In addition, BCIs enable to detect pre seizure early‐warning signals in EEG and then apply electrical stimulation to avoid or suppress the seizure.^22^ For BCI related to neural modulation, it may be necessary to consider using invasive methods, high‐quality data training modes within the safe and effective period of implantation, and the learning situation of the human brain itself in feedback training, in order to develop optimized modulation plan.

## 3 BCI PRESCRIPTION DEMONSTRATION FOR FUNCTIONAL DISORDERS

Although BCI can currently be used in the diagnosis, treatment, and rehabilitation of nervous system disease, the future BCI digital prescription for the needs of every individual patient must consider what their damaged functions are as the most important factor, to decide the design for BCI should focus more on its efficiency or stability. Based on these, choose which interaction paradigm to use, design corresponding encoding and decoding modes, and combine other technical outputs such as muscle functional electrical stimulation and brain electrical and magnetic stimulation to assist in functional rehabilitation. As shown in Table 1, the BCI digital description demonstration for various functional disorders should contain the Brain Signal, Interface Paradigm, and assistant technology.

**TABLE 1:** Description for various functional disorders.

Disorder	Disease	Requirement	Signal	Paradigm	Interface
Speech	ASL Stroke	Communication^9^, ^10^, ^17^, ^18^, ^23^	EEG	P300^9^	Text
				SSVEP^10^	Voice
			ECoG	Imagine speech/writing^17^, ^18^, ^23^	
Motor	ASL	Communication	EEG	MI^24^, ^25^, ^26^	Cursor
	Stroke	Rehabilitation^24^, ^25^, ^26^	ECoG		Smart home
					FES
					Exoskeleton
Consciousness	DOC	Evaluation^27^	EEG	P300 + SSVEP^27^	
		Communication^27^, ^28^, ^29^, ^30^		MI^28^, ^29^, ^30^	VT
Cognition	AD	Evaluation^31^	EEG	AEP	AR
		Modulation^20^		VEP	
Emotion	MMD	Evaluation^32^, ^33^, ^34^	EEG	Emotional^34^, ^35^, ^36^	TMS
	PDD	Modulation^35^, ^36^, ^37^, ^38^	ECoG		tDCS
	PSD				DBS
Visual	Hemianopia	Evaluation^39^	EEG	VEP^39^	tDCS
	Amblyopia	Restorations^40^	ECoG		ICMS
Auditory	Deafness	Evaluation^41^	EEG	AEP^41^	
Sleep	Sleep Apnea	Evaluation^42^	EEG	AEP^42^	ICMS

### 3.1 Communication BCI for speech disorders

The emergence of BCI technology has brought direct communication to patients who cannot express themselves through speech or physical movements. BC1s technology can not only serve as a tool for patients to communicate with the outside world but also improve neuronal plasticity by stimulating language circuits, thereby helping to treat aphasia. In the BCI digital prescription for Speech and language impairment, it is necessary to evaluate whether the patient is suitable for the passive spelling system or the active output mode.

Traditional communication control is based on a combination of motion imagination or one or more of P300 or SSVEP, passively implementing typing tasks. In 2017, BCI successfully helped three patients with post‐stroke residual dysfunction accurately control the cursor on the computer screen through simple imagination. Among them, patients were able to input an average of 39 letters 24 within 1 min.^17^ The use of hybrid BCI combined with visual steady‐state evoked potential and P300‐based BCI can improve overall communication performance.

More and more research using active interaction methods to achieve communication control, such as using speech imagination based BCI systems to design encoding and decoding modes related to pronunciation for continuous recognition of pronunciation intentions, and further synthesizing them into artificial speech. Chang et al. placing an electrode array in the auditory sensory cortex of paralyzed aphasia patients, deep learning algorithms automatically recognize the words the patient wants to say with 90 characters per minute by representing the part of the brain that produces phonemes, an area called the dorsal laryngeal cortex that is not yet well defined.^19^ And a 36‐year‐old male patient has learned to use brain activity to change the frequency of sound with two microelectrode arrays in the precentral gyrus and superior frontal gyrus. He can increase or decrease the frequency of sound waves to make the computer play fast or slow beeps, giving them the meaning of “yes” or “no”, then make a choice for a single letter and complete the free spelling, on the 107th days after BCI implantation, the patient “spoke” an unfamiliar German sentence.^23^

### 3.2 Rehabilitation BCI for motor dysfunction

People who have experienced spinal cord injury (SCI), amyotrophic lateral sclerosis (ALS), or stroke, usually lead to movement disorders and cannot independently complete daily tasks, such as communication, reaching out, and grasping. The main needs of people with motor dysfunction are to control the movement of the affected limbs or to control external assistive devices or home environments to alleviate the inconvenience of daily life. Kubler et al.^10^ confirmed that in patients with lock‐in syndrome caused by ALS, the goal of communicating with the external world is achieved by training them to control the movement of the cursor on the display screen. In four patients with severe bilateral upper limb paralysis,^24^ 16 electrode‐sensing devices were implanted into the superior sagittal sinus through the jugular vein. The nearby Precentral gyrus generates signals, and the motion signals are transmitted to the transceivers implanted under the clavicle through vascular wires to realize intention recognition.

On the other hand, it is more important to promote the rehabilitation process of limb function through the use of BCI and other technologies such as functional electrical stimulation. There are many studies on upper limb rehabilitation training in the current BCI system. Pfurtscheller et al.^25^ will be based on μ. The BCI Functional Electrical Stimulation (BCI–FES) system is applied to patients with quadriplegia. Through the BCI, activation control of upper limb electrical stimulation was achieved, enabling them to grasp the water cup and drink water, significantly improving the patient's upper limb function.

Moreover, the new type of microelectrode array can obtain higher precision action intentions, thereby providing more accurate control training for muscles or external devices. Schwemmer et al.^26^ implanted the microelectrode array into the left primary motor cortex of the patient in the area responsible for hand movement to control the functional electrical stimulation system on the surface of the affected limb, so that people with quadriplegia due to spinal cord injury can control the affected limb to complete the grasping action. BCI–FES training can stimulate neural plasticity and improve motor ability.

### 3.3 Evaluation of BCI for disorders of consciousness

For patients with Disorders of consciousness, BCI can provide an opportunity to confirm the existence of their consciousness. It is not only a method to evaluate consciousness but also a means to rebuild communication channels.

On the one hand, BCI is used to measure whether patients with DOCs have command following and response ability, and can be used as an auxiliary tool for clinical behavior evaluation. A study evaluated the visual localization of BCIs in 15 patients with DOCs and found that BCIs can overcome the shortcomings of the coma recovery scale, it is recommended to use BCIs as an auxiliary behavioral evaluation tool for detecting consciousness levels in the scale. Pan et al.^27^ proposed mixed auditory and visual BCIs based on auditory and visual evoked potentials. By displaying photos of healthy subjects and DIC patients simultaneously, participants were asked to select the corresponding photos according to instructions. The results showed that patients could detect command tracking, and the BCIs system could serve as a supportive bedside tool to detect the consciousness of patients with DOC.

In addition, researchers^28^ confirmed in their study on the application of BCI in patients with extremely low consciousness states that in the absence of other communication methods, it is possible to learn to use BCI for communication. Monti et al.^29^ took the lead in studying the possibility of BCIs system detecting compliance responses through the exercise imagination paradigm, and the results showed that patients with vegetative states were able to perform exercise imagination as instructed in fMRI experiments. In Monti et al.'s study,^30^ four out of 23 patients with unresponsive arousal syndrome underwent cognitive motor dissociation using functional magnetic resonance imaging to achieve communication. Cruse et al.^29^ used EEG based BCIs to collect EEG signals from 16 patients with vegetative state, and found that three patients could repeatedly and reliably follow instructions to imagine holding their right hand and imagining finger or toe movements.

### 3.4 Enhancement BCI for cognitive impairment

For patients with cognitive impairment, more needs are assessment monitoring and functional training. In the detection of cognitive impairment, the visual and auditory Oddball paradigms are used to obtain indicators such as P300 and MMN to evaluate their cognitive function. These characteristics and spontaneous state EEG rhythm can be used to monitor early cognitive impairment. The passive BCI has important clinical applications for Nervous system disease patients such as schizophrenia or depression. While the active BCI paradigm can also be used to achieve real‐time feedback, with different interaction modes designed for cognitive function enhancement training includes attention, memory.^19^, ^31^

### 3.5 BCI modulation for emotional dysfunction

Passive BCIs can be used to estimate emotional states,^32^, ^33^, ^34^ and enable diagnose several neural disorders, including depression,^35^ schizophrenia,^36^ and Parkinson's disease.^37^ For these patients, in addition to monitoring their emotional state, the closed‐loop BCI system can also regulate their emotional state through real‐time monitoring and predictive coding. Recent studies^38^ have promoted the optimization of TMS treatment regimens for depression, and found significant differences in the number of responders between the true stimulus group and the sham stimulus group. However, building a real‐time emotional warning system is different from existing research on monitoring abnormal discharges such as epilepsy. Emotional function is more regulated by sustainable rhythmic states, and emotion networks involving a large range of brain regions, it is complicated to find precise features to match the behavioral classification labels of emotion.

### 3.6 Restoration BCI for visual impairment

On the one hand, visual restoration can generate a sense of light by stimulating the responsive position on the brain. Daniel Yoshor^39^ uses implanted electrodes to apply stimuli to the surface of the human visual cortex in a dynamic order. When we use electrode stimulation to dynamically “draw” letters directly on the brain of blind people, they can “see” the corresponding letter shape and correctly recognize different letters. On the other hand, image recognition technology is used to decode neural signals and reconstruct the information seen. He et al.^40^ combined multi modal image feature semantic features with neural signal matching to achieve visual reconstruction, has proven that decoding new visual categories from human brain activities can be achieved with high accuracy.

### 3.7 Substitution BCI for hearing impairment

Cochlear implants can be considered as the earliest generalized BCI technology. The auditory paradigm for auditory and speech rehabilitation evaluation of cochlear implants provide more accurate reference basis, which has been applied to the evaluation of nearly 100 3–7‐year‐old cochlear implant children. Analyzing the auditory evoked potentials, it was found that the component P1 began to appear in the third month after implantation, but did not reach its peak until the sixth month. However, the temporal lobe was activated 3–6 months after implantation, and after approximately 12 months, the MMN waveform was basically normal.^41^

### 3.8 Modulation BCI for sleep disorder

Using the BCI to assist sleep staging can not only monitor sleep stages and sleep apnea events but also real‐time monitoring results can be used to make decisions to give certain interventions.^42^ Using certain frequency of stimulation to extend deep sleep, BCI can not only be used as evaluation but also as an intervention means for sleep disorder.

## 4 CLINICAL BCI EFFICACY EVALUATION

### 4.1 Communication indicators

As a communication system, information transmission rate of BCI is important performance indicator, it also depends on the number, accuracy, and efficiency of targets. However, the number and accuracy of targets, as well as transmission efficiency, are mutually constrained. In practical applications, while considering system performance, there is also a need to have a certain emphasis on balancing indicators related to specific requirements, different application scenarios have different requirements for real‐time performance and accuracy.

### 4.2 Neural ergonomic factors

Whether it is the active paradigm or the passive paradigm, as a human–computer interaction system, there is inevitably a certain degree of psychological and physical burden during the operation process, and real‐time accuracy and efficiency will be reduced due to fatigue. In system design, the impact of fatigue factors on real‐time changes in physiological signals caused by performance degradation and reception state interference should be considered. These human factors include system training time, performance, and workload, which can be measured by indicators related to availability and stability. In addition, it is also necessary to enhance the generalization and Transfer learning capabilities of the algorithm and design personalized training programs and feedback modes to improve the long‐term performance of the BCI system.

### 4.3 Functional recovery assessment

Many physical parameters may change with the progress of intervention of drugs or other therapies, especially for EEG which is more vulnerable to environmental interference. Spontaneous and evoked EEG signals during BCI training can be used as assessment of brain functional, and the behavior performance in BCI system also can be a prediction for intervention effect, by matching the functional recovery assessment method in clinical.

## 5 SUMMARY

In addition to considering the mechanisms of diseases and the specificity of patient brain signals, future BCI prescriptions also need to determine the classification of BCI digital prescriptions and the parameters of each module based on the evaluation results of patient perception, cognition, or motor injury, including the depth and density of collected signals, the interface content of interaction examples, and the encoding, decoding, and feedback modes of auxiliary peripheral modules. BCI is a means of neural rehabilitation training and regulation, as well as a means of evaluating functional status. It can also determine the next diagnosis, treatment, and rehabilitation training plan based on its ability to use BCI, and further combine with other intervention methods to help functional recovery.

The application of BCI technology in clinical practice, whether in neural rehabilitation or neural regulation, still has a certain distance to go. On the one hand, there are still some areas that need to be broken through in BCI technology, such as the safety of long‐term electrode implantation, the efficiency of real‐time decoding algorithms, and the stability of external device feedback. On the other hand, methods for evaluating the effectiveness of BCI‐assisted diagnosis or treatment for different diseases or functional impairments still need to be established; In addition, clinical trials and ethical standards for new medical devices are also under construction. Accelerating the application of BCI in clinical practice requires more researchers from interdisciplinary fields to work together.

## AUTHOR CONTRIBUTIONS

X.C. wrote the manuscript, T.C. Q.H., and N.W. sorted the references, X.S. and X.Z. checked the manuscript, Z.L., W.T., Y.Y., and J.Z. reviewed the manuscript.

## CONFLICT OF INTEREST STATEMENT

The authors declare that there are no conflicts of interest.

## Data Availability

Data sharing not applicable to this article as no datasets were generated or analysed during the current study.
